# Characteristics of Desertification and Short-Term Effectiveness of Differing Treatments on Shifting Sand Dune Stabilization in an Alpine Rangeland

**DOI:** 10.3390/ijerph16244968

**Published:** 2019-12-06

**Authors:** Xiao Feng, Jianjun Qu, Qingbin Fan, Lihai Tan, Zhishan An

**Affiliations:** 1 Dunhuang Gobi and Desert Research Station, Northwest Institute of Eco-Environment and Resources, Chinese Academy of Sciences, Lanzhou 730000, China; fengxiao@lzb.ac.cn (X.F.); fanqingbin@lzb.ac.cn (Q.F.); tanlihai09@lzb.ac.cn (L.T.); an1986wen@163.com (Z.A.); 2 University of Chinese Academy of Sciences, Beijing 100049, China; 3 Breeding Base for State Key Laboratory of Land Degradation and Ecological Restoration in Northwest China, Ningxia University, Yinchuan 750021, China

**Keywords:** vegetation planting, checkerboard protection, dune topographic locations, particle size distributions, soil nutrients, vegetation coverage

## Abstract

Rangeland desertification is one of the most serious problems threatening the ecological environment and socio-economic development on the eastern Qinghai-Tibet Plateau in China. To combat desertification and reduce its adverse effects, some strategies have been undertaken to stabilize the mobile sand dunes and restore the desertified land. In this study, rangeland desertification with a gradient degree of none, light, medium, severe and extreme was assessed, and short-term effectiveness of different treatments on stabilizing the shifting sand dunes was evaluated by monitoring selected vegetation and soil properties. Results showed that vegetation became thinner and sparser, and soil environment deteriorated significantly under desertification, leading to a poor and low diversity ecosystem. Applying a checkerboard protection strategy in which herb species were planted and using a shrub vegetation planting method without checkerboard protection on mobile dunes for five years, vegetation growth state and soil properties were improved. Soil particles were finer, vegetation restoration was more rapid, and soil nutrient improvement was more apparent at the lower locations of the sand dunes under the checkerboard protection planted with herbs, which performed slightly better in improving soil properties than the shrub planting method alone. A longer time period would be required for vegetation and soils on the sand dunes to be restored to sustain more intensive land use. These findings provide more insight into dune stabilization, allowing effective management in the ecological restoration of desertified rangeland.

## 1. Introduction

Aeolian desertification, a form of land degradation, is characterized by wind erosion in arid, semiarid and parts of the sub-humid regions in northern China and is mainly induced by excessive human activities [1,2]. Alpine regions on the Qinghai-Tibetan Plateau (QTP) are considered to be a unique geographical unit that is fragile and sensitive to environmental changes, due to the high elevation and cold climate [3]. If not managed properly, rangelands in these fragile alpine regions can easily become desertified. Rangeland desertification in these regions commences with the degradation of grassland or marshland, and continues towards an extremely serious state (e.g., mobile sand dunes), considered the final stage of land degradation [4,5]. Climate warming, decreasing precipitation, overgrazing, rodent damage, and repeated freezing-thawing actions are thought to be the primary driving factors responsible for the expansion of rangeland desertification on the eastern QTP [6,7,8,9,10].

Desertification adversely affects the survival and growth of rangeland plants and crops, due to strong wind erosion and mobile sand dune movement, threatening pastoral production and inhabitants’ living conditions [11]. Comprehensive research has been directed towards the control of aeolian desertification in China during the last 60 years [12]. Mobile sand dunes, generally 10–25 m high, are mainly composed of sand particles which lack cohesion, have high hydraulic conductivity, and are free of vegetation, making wind erosion one of the main limiting factors for plant growth [13]. Engineering measures, such as stone, straw or nylon ‘checkerboard’ barriers which are a type of vertical barrier with a certain size that serve as wind breaks, can be used to reduce wind-blown activity and facilitate sand stabilization. For example, straw checkerboards with a size of 100 cm long × 100 cm wide × 20 cm high, have been widely used to slow down the movement of sand particles and stabilize the shifting dunes in northern China, as they can effectively decrease wind speed near the dune surface [12,14,15]. Establishment of artificially planted vegetation in desertified areas is another effective method to control desertification and as a consequence improve the regional environment in northern China [16,17,18,19]. Enclosure treatments have been used to restore moderately and lightly degraded grasslands by prohibiting grazing, so contributing to the regeneration of vegetation and the reduction of wind erosion [20,21]. However, rangeland degradation in a desert state is difficult to restore naturally, as the rangeland abiotic conditions have been profoundly modified and the capacity of self-recovery within the rangeland ecosystem is limited [4]. As a result, it is difficult for rangeland vegetation to revert to its initial state, even if grazing exclusion is conducted. Therefore, a combination of different artificial input measures is necessary. Many studies have shown that the combinations of two or three treatments selected from the establishment of straw checkerboards, artificially planted vegetation and grazing exclusion have improved topsoil properties and facilitated vegetation restoration, thus are the most promising methods for sand fixation and ecological restoration in semiarid desertified lands [11,15,22,23].

Soil properties and vegetation indexes are important indicators to assess rangeland desertification and effectiveness of restoration strategies in alpine rangelands, as some literature suggests [23,24,25,26,27]. When assessing the short-term effectiveness of restoration, it might be more important to select some soil indicators that could reflect soil dynamics properties, such as soil organic matter, available phosphorus, nitrogen, and other nutrients [5]. Soil particle size distributions, soil bulk density, and soil water retention, which change slowly with time, could reflect long-term variations of soil qualities, thus could be used as indicators to assess rangeland desertification [28].

During the past decade, most desertification research has been concentrated in northern China where there is higher air temperature and lower precipitation than on the QTP. In addition, features of rangeland degradation (but not to a state of desertification), and short-time or long-time effectiveness of grazing exclusion on rehabilitating the degraded rangelands have been widely studied on the eastern QTP. However, features of rangeland desertification and methods for controlling the mobile sand dunes, have been rarely studied on the eastern QTP. Shifting sand dunes lead to sand storms, the loss of land resource, and reductions in land productivity, thus posing negative effects on sustainable regional social-economic development by threatening human health, destroying road and railway networks, and forcing people to vacate traditional lands [29]. It is crucial to understand the features of rangeland desertification to combat and control the shifting dunes, and it is necessary to evaluate whether these methods are effective and viable. Therefore, the main objective in this study is to assess the features of desertification and short-term effectiveness of different treatments to control mobile sand dunes in an alpine rangeland on the eastern QTP, using selected vegetation and soil property indices.

## 2. Materials and Methods

### 2.1. Study Area Description

**Figure 1:**
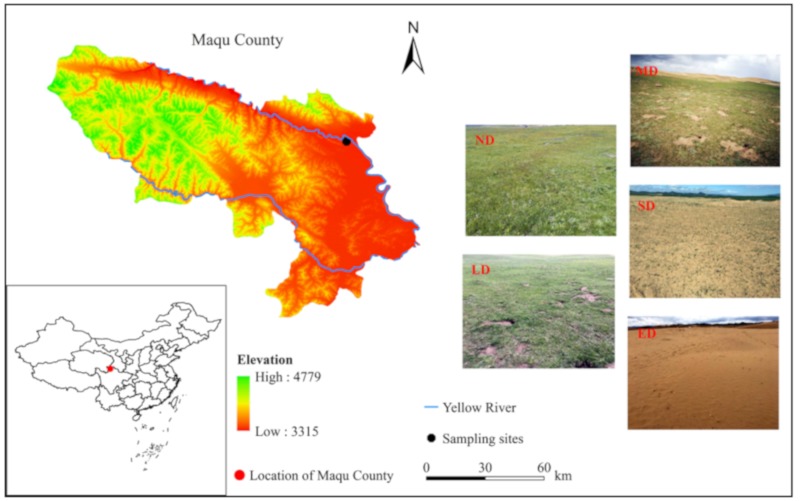
Location of the study area and landscapes of none desertified rangeland (ND), desertified rangelands: lightly (LD), medium (MD), severely (SD), and extremely (ED).

This study was carried out during July 2018 in one of the most typical desertified rangelands in Maqu county on the eastern QTP, where the elevation ranges from 3315 to 4779 m, as seen in Figure 1. The sampling sites are located at an elevation of 3500 m. Annual mean air temperature is 1.7 °C, precipitation is 600.4 mm/year, evaporation is 1274.1 mm/year, and sunshine duration is 2574.5 h/year, respectively. The average annual wind speed is 2.3 m/s, and the maximum wind speed is 20.7 m/s. Vegetation gross primary productivity is very low because of the cold climate. The vegetation type is dominated by alpine meadow, and sandy soil is widely distributed with a pH ranging from 7.0 to 7.5 (neutral to slightly alkaline).

### 2.2. Experimental Sampling Design and Analysis

Soil samples and vegetation data for assessing rangeland desertification were obtained from five desertification gradient sites, which were labelled as none (ND), lightly (LD), medium (MD), severely (SD), and extremely (ED) desertified rangeland, based on observed vegetation coverage. The landscapes of the five sampling sites are presented in Figure 1. The dominant plant species in the ND, LD, MD and SD sites were *Kobresia setchwanensis*, *Kobresia setchwanensis*, *Carex moorcroftii* and *Ephedra minuta*, respectively.

**Figure 2:**
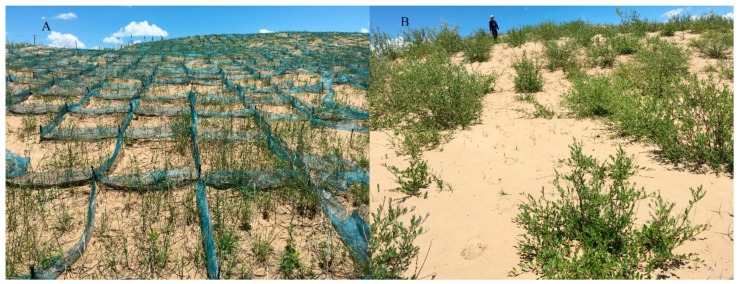
Sand fixing treatments on the shifting dunes. (**A**) represents the checkerboard protection planted with herbaceous vegetation, and (**B**) represents the shrub vegetation planting method.

Treatments for stabilizing the shifting sand dunes in our study included: (a) checkerboard protection planted with *Elymus nutans* (CE); (b) checkerboard protection planted with *Medicago sativa* (CM); (c) shrub vegetation planting (*Salix oritrepha*) without checkerboard protection (S), as shown in Table 1 and Figure 2. The checkerboard framework was made up of nylon sand barriers, 100 cm long × 100 cm wide × 20 cm high. The plants *Elymus nutans* and *Salix oritrepha* are widespread native species, while *Medicago sativa* is an introduced edible herbaceous species.

After stabilizing the shifting sand dunes for five years, soil samples and vegetation data for evaluating the effectiveness of each method were collected along the windward slope of the bottom (BOT), middle (MID) and top (TOP) of sand dunes treated with CE, CM and S, respectively. Soil samples collected from the adjacent bare sand dunes were used as the control treatment (CK).

At each sampling site, five random quadrat plots were surveyed for vegetation and three sampled for soil. The quadrat plots were 0.5 m × 0.5 m for herb study in the desertified rangeland sites, 2 m × 2 m for shrub treatment, and 1 m × 1 m for herb treatment on the mobile sand dunes. All soil samples were collected from 1 m × 1 m quadrat plots. In each quadrat plot, vegetation coverage, plant height and dried aboveground biomass were recorded for herbs, and plant density, height and canopy were recorded for shrubs.

Soil properties could properly reflect variations under desertification within the uppermost 30 cm; however, to reflect variations in soils on sand dunes under short-term stabilization effectively, it could be better for us to collect soil samples on the dune top surface but not to a deeper depth, as soil degradation and restoration are both very slow processes [17,30,31]. Therefore, soil samples for analyzing soil particle size distributions (PSDs) were collected from soil layers of 0–5, 5–10, 10–20 and 20–30 cm in the desertified sites, and from the 0–5 cm soil layer in the sites under stabilization treatments. Soil samples for analyzing soil organic carbon (SOC), total nitrogen (TN), available nitrogen (AN), available phosphorus (AP), and available potassium (AK) were collected from soil layers of 0–30 cm in the desertified sites, and from the 0–5 cm soil layer in the sites under stabilization. Soil samples in a given layer were collected from 8–10 points and mixed homogeneously to obtain a composite sample. Soil samples for analyzing soil water content (SW) and soil bulk density (BD) were collected from soil layers of 0–10, 10–20 and 20–30 cm in the desertified sites using a stainless-steel cutting ring with a volume of 100 cm^3^.

The aboveground portions of herbaceous plants were cut flush with the ground. Plant samples were rinsed with water and dried in a constant-temperature oven at 85 °C until constant weight was attained [23]. Vegetation coverage was assessed by the vertical projection method [15,22]. Plant canopy size was determined by the average value of the east-west and north-south length of the shrub.

Soil samples were air dried and hand sieved through 2 and 0.5 mm sieves to remove roots and other debris. Soil particle sizes were measured by a laser diffraction particle-size analyzer (mod. 2000, Malvern Instruments, Malvern, UK) with a measurement range of 0.02–2000 μm and a repeated measurement error of less than 2%, thus yielding the volumetric distributions of soil PSDs [32]. Following the classification system from the United States Department of Agriculture [17,20], we measured the particle sizes in 5 classes: <2 (clay), 2–50 (silt), 50–100 (very fine sand), 100–250 (fine sand) and 250–500 μm (medium sand). SOC was determined by the K_2_Cr_2_O_7_-H_2_SO_4_ oxidation method. Soil TN was analyzed using the Kjeldahl digestion procedure. Soil AN was determined using the continuous alkali-hydrolyzed reduction diffusion method. Soil AP was measured using the ultraviolet spectrophotometer method. Soil AK was determined by the flame photometer method [32]. SW and soil BD were determined by soil weight difference before and after oven-drying at 105 ℃ for 8 h [20]. The SW contents and soil BD at the soil layer of 0–30 cm were calculated by the average contents of those at the 0–10, 10–20 and 20–30 cm layers.

### 2.3. Data Analysis

A one-way ANOVA was used to analyze the effects of rangeland desertification, sand stabilization treatments and dune topographic positions on each vegetation index and soil property. The general liner model was used to evaluate the effects of sand stabilization treatments, dune topographic locations and interactions of sand stabilization treatments × dune topographic locations on soil properties. The Least Significant Difference (LSD) procedure was applied to separate the means at a significance level of *p* < 0.05. The results were expressed as mean values ± standard error (SE). Pearson correlation analysis was applied to investigate the relationships among the soil properties. Data in this study were analyzed by the software of IBM SPSS Statistics 19.0 from Chicago, USA.

## 3. Results

### 3.1. Features of Rangeland Desertification

#### 3.1.1. Selected Vegetation Traits under Rangeland Desertification

**Figure 3:**
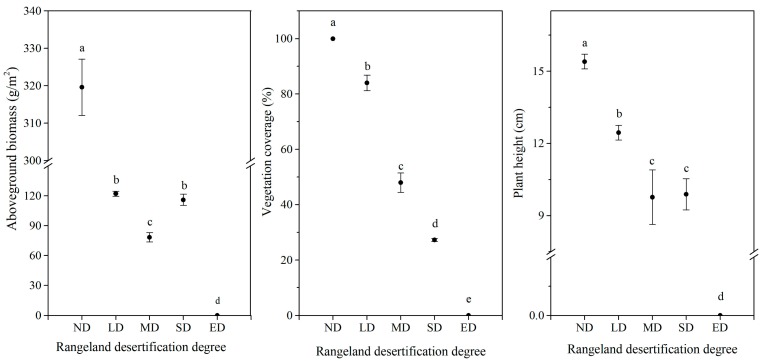
Selected vegetation indexes under five degrees of desertification. Vertical bars indicate standard errors of means (*n* = 5). Different letters indicate significant difference of each vegetation index among the desertification degrees at the 0.05 level.

Plant coverage, biomass and height are important indicators for evaluating the extent of vegetation degradation and restoration [11]. As seen in Figure 3, with increasing rangeland desertification, the aboveground biomass, vegetation coverage and plant height decreased significantly (*p* < 0.05, the SD was excluded in terms of the aboveground biomass and plant height). The aboveground biomass of the SD with lower vegetation coverage was larger than that of the MD, and showed no significant difference with that of the LD with a larger vegetation coverage. This was because the dried aboveground biomass of *Ephedra minuta* was larger than that of *Carex moorcroftii* and *Kobresia setchwanensis*, due to their physiological differences. The results indicate that desertification leads to thinner and sparser vegetation cover.

#### 3.1.2. Characteristics of Soil Particle Size Distributions under Rangeland Desertification

**Figure 4:**
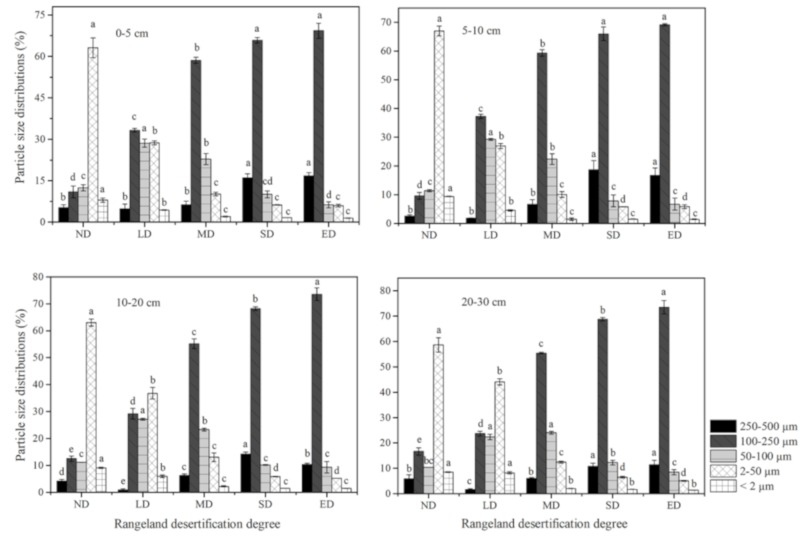
Variations in soil particle size distributions under five degrees of rangeland desertification. Vertical bars indicate standard errors of means (*n* = 3). Different letters in the same symbol indicate significant difference of the proportion of each particle size class among the desertification degrees at the 0.05 level.

With increasing desertification (from ND to ED) at each soil layer, the contents of clay and silt particles decreased significantly (*p* < 0.05), and the fine sand content increased significantly (*p* < 0.05). From LD to ED at each soil layer, the very fine sand content decreased significantly (*p* < 0.05), while the medium sand content increased significantly (*p* < 0.05) (Figure 4). The characteristics of the soil PSDs showed that the clay, silt and very fine sand particles were selectively removed, while fine sand and medium sand particles were accumulated, indicating that soils were progressively coarsened with increasing desertification degree.

#### 3.1.3. Selected Soil Properties under Rangeland Desertification

Selected soil properties, including SOC, soil TN, AN, AP, AK, BD and SW at five degrees of rangeland desertification from the 0–30 cm soil layer, are shown in Table 2. The results indicated that with increasing desertification degree from ND to ED, the contents of SOC, TN, AN, AP, AK and SW decreased significantly (*p* < 0.05), while soil BD increased significantly (*p* < 0.05).

### 3.2. Effectiveness of Treatments on Shifting Sand Dune Stabilization

#### 3.2.1. Selected Vegetation Indexes under Sand Stabilization Treatments

It is noted that the dominant plant species was found to be *Elymus nutans* at the middle and top locations, and *Kobresia setchwanensis* at the bottom location on the sand dune treated with CM. During the dune fixing process, *Medicago sativa*, artificially planted on the dune surface, died gradually and was replaced by *Elymus nutans* and *Kobresia setchwanensis*.

As seen from Table 3, along the slope of the dunes from the bottom, middle to top under the treatments of CE and CM, the aboveground biomass and vegetation coverage decreased significantly (*p* < 0.05). The plant height under the CE treatment showed a decrease from the bottom to top. The plant height was 17 cm at the bottom location, where the dominant plant species was *Kobresia setchwanensis*, while the height was 33 and 25 cm at the middle and top positions of the sand dune treated with the CM, respectively, where the dominant species for both was *Elymus nutans*. The shrub height and canopy at the bottom and top locations were significantly larger than those at the middle position under the S treatment. The results indicated that the CE and CM treatments greatly facilitated vegetation growth at the lower positions, while the S treatment improved the plant growing state at the top and bottom locations of the sand dunes.

#### 3.2.2. Characteristics of Soil Particle Size Distributions under Sand Stabilization Treatments

Table 4 shows variations in soil particle size distributions on the mobile sand dunes at three positions under four treatments at 0–5 cm soil depth. The predominant particles were fine sand, ranging between 60% and 74% of total volume. Differences among the treatments in terms of the clay, silt, very fine sand, and medium sand contents were significant (*p* < 0.05), as seen from Table 5.

Compared to CK, the clay and silt contents were higher under the treatments of CE and CM, and the very fine sand contents were higher and the medium sand contents were lower under the CE, CM and S treatments, indicating that these sand stabilization treatments contributed to a fine soil texture. The clay and silt contents were higher under the CE treatment than those under the S treatment, indicating that the checkerboard protection planted with herbs was more prone to trapping fine soil particles.

Differences among the dune topographic positions in terms of the silt, very fine sand, and fine sand contents were significant (Table 5). The silt and very fine sand contents decreased, while the fine sand contents increased significantly along the slope from the bottom to top of the sand dunes treated with CE and CM, indicating that soil particles were finer at the lower positions of the dunes. However, differences were not significant among the bottom, middle and top locations of the dunes under the CK and S treatments for soil PSDs.

#### 3.2.3. Selected Soil Properties under Sand Fixing Treatments

**Figure 5:**
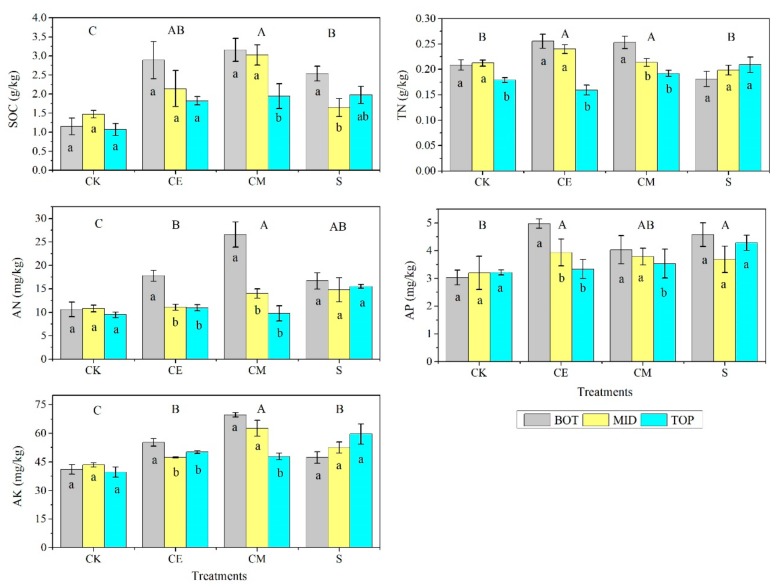
Selected soil properties within the 0–5 cm soil layer at three positions of the mobile sand dunes under four treatments. Different lowercase letters indicate significant difference at the 0.05 level among the positions under the same treatment, and different uppercase letters indicate significant difference at the 0.05 level among the treatments, in terms of each soil property.

Differences among the treatments of CK, CE, CM and S, in terms of each soil property, were significant (*p* < 0.05), as seen from Table 6. The SOC, AN and AK contents under the treatments of CE, CM and S, the TN contents under the CE and CM, and the AP contents under the CE and S, were higher compared to CK (Figure 5). The SOC and TN contents under the CE and CM treatments were higher than those under the S treatment, and differences were significant between the CM and S. The AK content under the CM was significantly higher than that under the S treatment. The results indicated that the three treatments, especially CM, contributed to improving soil properties after stabilizing the mobile sand dunes for 5 years.

Differences among the dune positions were significant (*p* < 0.05), in terms of SOC, TN and AN contents (Table 6). Results showed that the contents of SOC, TN and AN were higher at the bottom locations than those at the top locations (data not displayed). As seen from Figure 5, the contents of the five soil indicators under the CE and CM treatments were decreasing, along the slope from the bottom to top of the sand dunes. However, differences were generally not significant among the dune positions under the CK and S treatments.

### 3.3. Correlations among Soil Properties

Relationships among the sand, silt and clay contents and selected soil properties from sample sites under desertification and stabilization are shown in Table 7. As seen, for sample sites under desertification, the SOC, TN, AN, AP, AK and SW contents were in significant positive correlation with the clay and silt contents and showed a very strong negative correlation with the sand contents, with coefficients ranging from 0.92 to 1. The BD were negatively correlated with the clay and silt contents, and positively correlated with the sand contents. The SOC, TN, AN, AP and AK contents had a strong positive correlation with the SW contents, but had a negative correlation with soil BD. In addition, the SW contents were significantly negatively correlated with soil BD.

Compared to the sampling sites under desertification, weaker relationships were found among the selected soil properties and the sand, silt and clay contents under the sand fixing treatments, with coefficients ranging from 0.41 to 0.81. The SOC, TN, AN and AP contents showed strong negative correlations with the sand contents and showed positive correlations with the silt and clay contents.

## 4. Discussion

### 4.1. Characteristics of Rangeland Desertification

Desertification resulted in decreased vegetation coverage, plant height and aboveground biomass. Li et al. [4] reported that in semi-arid steppe on the QTP, rangeland vegetation coverage decreased continuously until desertification occurred. Overgrazing, one of the main driving factors for rangeland desertification, depletes soil nutrients continuously, which in turn affects vegetation coverage and biomass adversely [4]. With decreasing vegetation coverage, bare soil areas increased, especially during the winter and spring when vegetation coverage is lower, soils are more easily exposed to windy environments without protection, and thus, fine soil particles such as clay and silt are preferentially eroded and removed by wind [33,34], resulting in coarser soil textures [35].

The present study showed that with increasing desertification degree, the SOC, TN, AN, AP, AK, and SW contents decreased significantly, while soil BD increased significantly, leading to a poor desert soil environment. The Pearson correlation analysis showed that the SOC, TN, AN, AP, AK and SW contents were in significant positive correlation with the clay and silt contents, and showed a very strong negative correlation with the sand contents. Previous studies have shown that the losses of SOC, TN and other soil nutrients were mainly due to the removal of fine particles (silts and clays) induced by wind erosion in dry land [21,35,36,37,38]. Compared to sand particles, silt and clay particles had higher capacity for holding soil water and nutrients [39,40,41]. In addition, decreased vegetation biomass and lower litter decomposition caused the reduction input of soil organic matters, thus, soil nutrients decreased accordingly [42]. Soil BD was negatively correlated with the clay and silt contents, and was positively correlated with the sand contents, mainly due to the reduction in soil organic matter contents and pore space [43,44,45].

### 4.2. Short-Term Effectiveness of the Treatments on Fixing Mobile Sand Dunes

The aboveground biomass and vegetation coverage decreased significantly from the bottom to top of the sand dunes under the CE and CM treatments, indicating that the checkerboard protection planted with herbs could facilitate vegetation restoration better at the lower parts of the dunes more than that at the upper parts. After the checkerboard barriers were established on the mobile sand dunes, the near-surface wind speed decreased and the sediment transport reduced, thus the shifting dune surface gradually stabilized, forming an environment with a weakened wind strength and providing favorable conditions for plants [46,47]. However, wind speed increases with increasing dune height, therefore, the upper parts of sand dunes have higher wind power, resulting in higher mobility of sand particles, stronger wind erosion and deposition than the lower parts. Thus, herbaceous plants and their seeds are more difficult to fix on the dune surfaces at the upper parts and were more easily blown away by wind erosion and buried by the accumulated sand particles [14]. In addition, the frequent windy days last from November to April when vegetation has stopped growing, and during the same period there was lower precipitation across the study area. This natural phenomenon causes stronger aeolian movement, due to the lower vegetation coverage and drier sandy soil environment on dunes, which in turn adversely affects vegetation growing environments.

During the plant restoration process, the alien plant species *Medicago sativa* was replaced naturally by two native plant species, mainly because the native species are best adapted to the local environments and survived. Therefore, native plant species was suggested to be used, when fixing mobile dunes.

The height and canopy of shrubs, at the middle location of the sand dunes under the shrub vegetation planting treatment, were significantly lower than those at the top and bottom locations. It was noted that the top site of the sand dune planted with shrub vegetation was less steep and flatter than the middle and bottom sites. Feng et al. [48] reported that wind erosion showed an increase with increasing surface slopes. Therefore, vegetation is more prone to wind erosion and deposition and was slower to restore at the middle sites with a steep slope. Some literature [31,49] also suggests that vegetation restoration is more rapid on flat sandy land and lower parts of sand dunes, while it takes longer on upper and steeper parts of the dunes.

Soil particles were finer, and nutrients had been improved under the CE, CM and S treatments, compared to the bare sand dune. The checkerboard treatment planted with herbs, and the shrub planting treatment without checkerboards contributed to trapping wind-blown fine particles (such as the very fine sand, silt, and clay particles), by decreasing surface wind speed and protecting soil particles from further entrapment [47]. This is very important for sand fixation, because fine particles adhering to each other may enhance the stability of the sand dune surface [50]. During the vegetation restoration process on dunes, vegetation increased soil organic matter input and soil water holding capacity, and reduced the losses of nutrients by wind erosion, thereby contributing to the accumulation of soil nutrients [16,19,39], which in turn favored vegetation restoration. In addition, the dead *Medicago sativa* on dunes under the CM treatment led to higher input of organic matters into soils.

The lower locations of the dunes under the checkerboard protection planted with herbs, contributed to improving soil nutrients and textures more than the higher locations did, mainly due to the larger vegetation coverage, higher soil water contents, and lower wind speed at the lower locations. As vegetation coverage decreased at the upper parts of dunes, sediment flux increased, and fine particles were more prone to removal by wind erosion, so soil textures were coarser, and nutrients were lower [47,51]. However, differences of soil properties were not significant among the dune positions under the S treatment, probably because shrubs were less efficient at trapping fine particles, and sand particles could move more easily due to the reduced roughness at the dune surface, relative to the checkboard protection planted with herbs [15]. This also explained why the checkboard protection planted with herbs improved soil properties on the dune surfaces slightly more than the shrub planting treatment alone did. Therefore, it is useful to establish the checkboard protection before applying the shrub planting method.

The CM, CE and S treatments contributed to trapping fine particles and improving soil nutrients on the dune uppermost surface and were effective at stabilizing the mobile dunes. Vegetation, at the top, middle and bottom locations of the shifting sand dunes under the CE and CM treatments, was restored to the levels of the ED, SD and MD sites, respectively. Meanwhile, soil SOC, TN and AN content on the dunes were restored to the level of the SD site, indicating slower soil restoration processes compared with the vegetation restoration. Miao et al. [22] also reported that soil restoration had a lag effect relative to vegetation restoration in degraded grasslands. Generally, the ecologically harsh environments (including low temperatures, windy environments, very low percentages of silt and clay, limited soil moisture, and infertile sandy soil) make vegetation and soil recovery on shifting sand dunes very slow, especially in alpine rangelands with a high elevation and cold climate. As some literature suggests [30,31,49], it would take a longer time for vegetation and soil properties to be satisfactorily restored on mobile sand dunes in order to sustain more intensive land use.

## 5. Conclusions

In this study, we assessed the features of desertification and short-term (5 years) effectiveness of three treatments on fixing mobile sand dunes on the windward slope by monitoring selected vegetation indexes (including aboveground biomass, vegetation coverage and plant height for herbs, and plant density, height and canopy for shrubs) and soil properties (including soil particle size distributions, soil BD, SW, SOC, TN, AN, AP and AK) in an alpine rangeland with an elevation of 3500 m on the eastern QTP.

With increasing desertification degree, aboveground biomass, vegetation coverage and plant height were significantly decreased. Soil textures were coarsened with the loss of clay, silt and very fine sand particles, and the accumulation of fine sand and medium sand particles; soil nutrients, (including SOC, TN, AN, AP and AK) reduced, soil water contents decreased, and soil BD increased significantly under increasing rangeland desertification.

The checkerboard protection planted with *Elymus nutans* and *Medicago sativa* (herbs), and the *Salix oritrepha* (shrub) planting method without checkerboards, are effective in restoring vegetation and improving soil textures as well as nutrients on the mobile sand dune surface. The checkerboard protection planted with herbs performed slightly better in improving soil properties than the shrub planting treatment alone. Aboveground biomass, vegetation coverage and plant height decreased, soil textures became coarsened, and soil nutrients decreased along the slope from the bottom, middle to the top locations of the dunes under the checkerboard protection planted with herbs. The shrub planting treatment improved the plant height and canopy size more at the top and bottom locations so than the middle location of the dunes, but had little effect on soil textures and nutrients on the three dune topographic locations. Native plant species are proposed for restoring vegetation in desertified rangelands, and a longer time period would be required for vegetation and soils to be restored sufficiently to sustain more intensive land use.

## Figures and Tables

**Table 1:** Five years treatments for fixing the mobile sand dunes.

Abbreviations	Sand Fixing Treatments	Vegetation Types	Vegetation Species	Number of Sand Fixing Years
CE	Checkerboard protection planted with herbaceous vegetation	Herb	*Elymus nutans*	5
CM	Checkerboard protection planted with herbaceous vegetation	Herb	*Medicago sativa*	5
S	Shrub vegetation planting method	Shrub	*Salix oritrepha*	5

CE—the treatment of checkerboard protection planted with *Elymus nutans*, CM—the treatment of checkerboard protection planted with *Medicago sativa*, S—the treatment of shrub vegetation planting method.

**Table 2:** Selected soil properties under five degrees of rangeland desertification within the 0–30 cm soil layer. Data are means ± SE (*n* = 3).

	SOC (g/kg)	TN (g/kg)	AN (mg/kg)	AP (mg/kg)	AK (mg/kg)	BD (g/cm^3^)	SW (%)
ND	111.4 ± 3.4 ^a^	8.17 ± 0.09 ^a^	936.5 ± 70.9 ^a^	9.6 ± 2.1 ^a^	120.0 ± 3.1 ^a^	0.71 ± 0.03 ^d^	42.2 ± 2.2 ^a^
LD	23.1 ± 0.8 ^b^	1.99 ± 0.06 ^b^	228.7 ± 4.3 ^b^	6.0 ± 0.2 ^b^	115.2 ±5.6 ^a^	1.23 ± 0.01 ^c^	18.8 ± 0.3 ^b^
MD	6.0 ± 0.4 ^c^	0.47 ± 0.03 ^c^	65.7 ± 5.5 ^c^	4.5 ± 0.2 ^b^	63.7 ± 2.5 ^b^	1.38 ± 0.04 ^b^	6.8 ± 0.5 ^c^
SD	1.6 ± 0.1 ^c^	0.21 ± 0.01 ^d^	15.8 ± 0.1 ^c^	3.4 ± 0.1 ^b^	45.2 ± 3.3 ^c^	1.57 ± 0.04 ^a^	4.1 ± 0.1 ^c^
ED	1.6 ± 0.1 ^c^	0.17 ± 0 ^d^	8.8 ± 0.2 ^c^	2.9 ± 0.5 ^b^	39.1 ± 1.7 ^c^	1.64 ± 0.01 ^a^	3.8 ± 0.2 ^c^

Different letters in the same column indicate significant difference of each soil property among the desertification degrees at the 0.05 level.

**Table 3:** Selected vegetation indexes at three topographic locations of the shifting dunes under three sand treatments. Data are means ± SE (*n* = 5).

Sand Fixing Treatments	Selected Vegetation Indexes	Topographic Locations of Sand Dunes		
		BOT	MID	TOP
**CE**	Vegetation coverage (%)	46 ± 0.7 ^a^	30 ± 1.9 ^b^	4 ± 0.4 ^c^
	Plant height (cm)	35 ± 1.3 ^a^	25 ± 2.5 ^b^	22 ± 0.6 ^b^
	Aboveground biomass (g/m^2^)	71 ± 2.5 ^a^	30 ± 2.6 ^b^	6 ± 0.6 ^c^
**CM**	Vegetation coverage (%)	47 ± 3.1 ^a^	30 ± 1.0 ^b^	6 ± 0.4 ^c^
	Plant height (cm)	17 ± 2.1 ^b^	33 ± 3.0 ^a^	25 ± 1.8 ^ab^
	Aboveground biomass (g/m^2^)	79 ± 1.8 ^a^	32 ± 3.2 ^b^	7 ± 1.3 ^c^
**S**	Plant density (Plants/m^2^)	0.32 ± 0.02 ^a^	0.35 ± 0.01 ^a^	0.30 ± 0.01 ^a^
	Plant height (cm)	150 ± 7.1 ^a^	90 ± 10.6 ^b^	177 ± 2.5 ^a^
	Plant canopy (cm)	148 ± 13.7 ^b^	90 ± 9.1 ^c^	209 ± 5.4 ^a^

Different letters in the same row indicate significant difference of each vegetation index among the dune topographic locations at the 0.05 level.

**Table 4:** Proportion of soil particles (%) within the 0–5 cm soil layer at three positions of the mobile sand dunes under four treatments. Data are means ± SE (*n* = 3).

Soil Particle Size Classes (μm)	Treatments	Positions of Sand Dunes			
		BOT	MID	TOP	Mean
<2	CK	1.1 ± 0.0 ^Cc^	1.4 ± 0.0 ^Ab^	1.2 ± 0.0 ^Ba^	1.2 ^c^
	CE	1.8 ± 0.1 ^Aab^	1.7 ± 0.0 ^Aa^	1.6 ± 0.1 ^Aa^	1.7 ^a^
	CM	1.9 ± 0.1 ^Aa^	1.3 ± 0.1 ^Bb^	1.6 ± 0.2 ^ABa^	1.6 ^ab^
	S	1.5 ± 0.1 ^Ab^	1.4 ± 0.1 ^Ab^	1.3 ± 0.1 ^Aa^	1.4 ^bc^
	Mean	1.6 ^A^	1.4 ^A^	1.4 ^A^	
2–50	CK	4 ± 0.3 ^Ac^	5 ± 0.3 ^Ab^	5 ± 0.4 ^Aa^	4.8 ^c^
	CE	9 ± 0.8 ^Aa^	8 ± 0.5 ^ABa^	6 ± 0.4 ^Ba^	7.5 ^a^
	CM	8 ± 0.2 ^Aa^	5 ± 0.2 ^Bb^	6 ± 1.0 ^Ba^	6.3 ^b^
	S	6 ± 0.6 ^Ab^	5 ± 0.5 ^Ab^	5 ± 0.4 ^Aa^	5.5 ^bc^
	Mean	6.8 ^A^	5.8 ^B^	5.4 ^B^	
50–100	CK	6 ± 0.5 ^Ab^	3 ± 1.5 ^Ab^	4 ± 1.6 ^Ab^	4.5 ^b^
	CE	14 ± 1.4 ^ABa^	15 ± 2.2 ^Aa^	9 ± 0.7 ^Bab^	12.6 ^a^
	CM	16 ± 1.5 ^Aa^	10 ± 1.5 ^Ba^	7 ± 1.0 ^Bb^	11.0 ^a^
	S	12 ± 1.9 ^Aa^	10 ± 0.7 ^Aa^	11 ± 1.0 ^Aa^	11.0 ^a^
	Mean	11.7 ^A^	9.7 ^AB^	7.9 ^B^	
100–250	CK	66 ± 1.4 ^Aa^	65 ± 1.0 ^Ab^	70 ± 3.6 ^Aa^	66.8 ^a^
	CE	60 ± 1.4 ^Ca^	65 ± 1.6 ^Bb^	74 ± 0.4 ^Aa^	66.5 ^a^
	CM	64 ± 1.1 ^Ba^	70 ± 0.5 ^Aa^	73 ± 1.5 ^Aa^	68.9 ^a^
	S	65 ± 3.3 ^Aa^	69 ± 0.9 ^Aa^	71 ± 1.3 ^Aa^	68.3 ^a^
	Mean	63.6 ^C^	67.4 ^B^	71.9 ^A^	
250–500	CK	22 ± 1.1 ^Aa^	25 ± 2.3 ^Aa^	20 ± 4.8 ^Aa^	22.6 ^a^
	CE	16 ± 2.2 ^Aab^	10 ± 1.9 ^ABb^	9 ± 0.2 ^Bb^	11.7 ^b^
	CM	11 ± 2.2 ^Ab^	14 ± 1.4 ^Ab^	12 ± 1.9 ^Aab^	12.3 ^b^
	S	16 ± 4.3 ^Aab^	14 ± 0.9 ^Ab^	11 ± 1.9 ^Aab^	13.6 ^b^
	Mean	16.1 ^A^	15.7 ^A^	13.4 ^A^	

Different uppercase letters in the same row indicate significant difference among the dune topographic locations, and different lowercase letters in the same column indicate significant difference among the treatments at the 0.05 level, in terms of the proportion of each soil particle size class.

**Table 5:** F and *p*-values of soil particle size distributions analyzed by the general liner model, in which T, P and T × P represent treatments, positions of sand dunes, and interactions of treatments × positions of sand dunes, respectively.

	250–500 μm		100–250 μm		50–100 μm		2–50 μm		<2 μm	
	F-Value	*p*-Value	F-Value	*p*-Value	F-Value	*p*-Value	F-Value	*p*-Value	F-Value	*p*-Value
T	13.014	<0.001	1.361	0.279	19.94	<0.001	15.51	<0.001	12.22	<0.001
P	1.465	0.251	22.87	<0.001	7.8	0.002	8.31	0.002	2.94	0.072
T × P	0.865	0.534	2	0.105	2.8	0.033	3.26	0.017	4.34	0.004

**Table 6:** F and *p*-values of selected soil properties analyzed by the general linear model, in which T, P and T × P represent treatments, positions of sand dunes, and interactions of treatments × positions of sand dunes, respectively.

	SOC		TN		AN		AP		AK	
	F-Value	*p*-Value	F-Value	*p*-Value	F-Value	*p*-Value	F-Value	*p*-Value	F-Value	*p*-Value
T	14.459	<0.001	3.988	0.019	11.359	<0.001	4.086	0.018	24.721	<0.001
P	6.626	0.005	15.775	<0.001	21.894	<0.001	2.414	0.111	2.211	0.131
T × P	1.889	0.124	7.300	<0.001	6.989	<0.001	1.227	0.327	7.711	<0.001

**Table 7:** Pearson correlation coefficients among selected soil properties under rangeland desertification and sand stabilization treatments.

	Sand	Silt	Clay	SOC	TN	AN	AP	AK	BD	SW
Sampling sites under rangeland desertification (*n* = 5)										
Sand	1									
Silt	−1.00 **	1								
Clay	−1.00 **	0.99 **	1							
SOC	−0.95 *	0.95 *	0.92 *	1						
TN	−0.96 **	0.96 **	0.93 *	1.00 **	1					
AN	−0.96 **	0.97 **	0.94 *	1.00 **	1.00 **	1				
AP	−0.99 **	0.99 **	0.97 **	0.96 **	0.97 **	0.97 **	1			
AK	−0.92 *	0.92 *	0.95 *	0.76	0.78	0.79	0.90 *	1		
BD	0.98 **	−0.98 **	−0.96 *	−0.96 *	−0.96 **	−0.97 **	−1.00 **	−0.89 *	1	
SW	−0.99 **	0.99 **	0.98 **	0.98 **	0.99 **	0.99 **	0.99 **	0.87	−0.98 **	1
Sampling sites under the sand stabilization treatments (*n* = 12)										
Sand	1									
Silt	−0.99 **	1								
Clay	−0.92 **	0.91 **	1							
SOC	−0.69 *	0.69 *	0.63 *	1						
TN	−0.64 *	0.66 *	0.45	0.55	1					
AN	−0.59 *	0.57	0.56	0.75 **	0.57	1				
AP	−0.69 *	0.69 *	0.55	0.73 **	0.47	0.61 *	1			
AK	−0.41	0.42	0.44	0.81 **	0.50	0.81 **	0.50	1		

** and * are significant at the 0.01 and 0.05 level (2-tailed), respectively.

## Abbreviations

QTP	Qinghai-Tibet Plateau
ND	None desertified rangeland
LD	Lightly desertified rangeland
MD	Medium desertified rangeland
SD	Severely desertified rangeland
ED	Extremely desertified rangeland
CE	The treatment of checkerboard protection planted with *Elymus nutans*
CM	The treatment of checkerboard protection planted with *Medicago sativa*
S	The treatment of shrub vegetation planting method
CK	The treatment of control
BD	Soil bulk density
SW	Soil water content
PSDs	Soil particle size distributions
